# Correction: Cdc42 interacts with chaperone Ydj1 to enhance its stability and partitioning during asymmetric cell division and aging in yeast

**DOI:** 10.1371/journal.pbio.3003891

**Published:** 2026-07-02

**Authors:** The PLOS Biology Staff

There are a number of errors in the caption for Fig 4, “Farnesylated Ydj1 is required for maintaining Cdc42 levels and its asymmetric distribution,” panels A-C. Please see the complete, correct Fig 4 caption here.

The publisher apologizes for the error.

**Fig 4:**
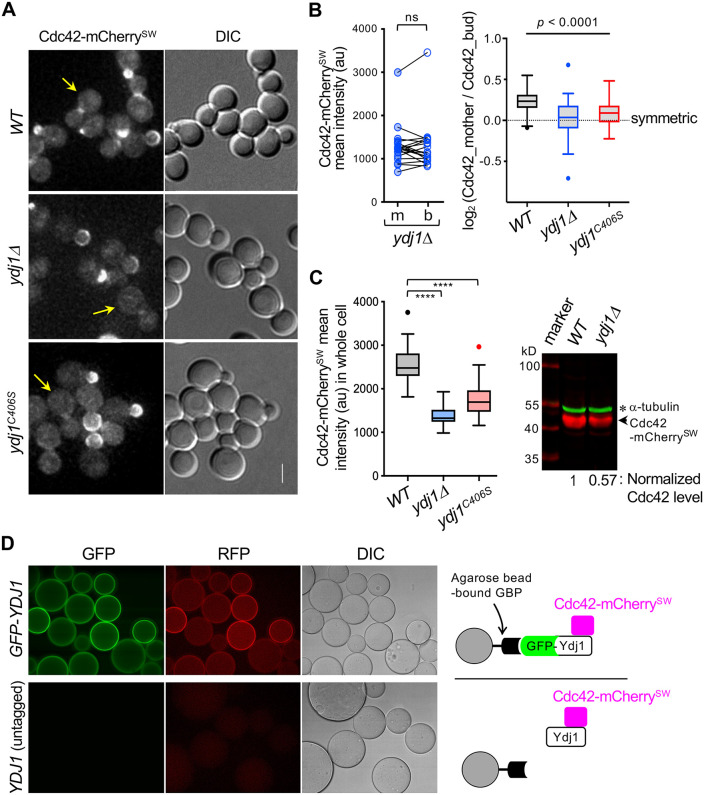
Farnesylated Ydj1 is required for maintaining Cdc42 levels and its asymmetric distribution. **A.** Localization of Cdc42-mCherrySW in WT and ydj1 mutants at 27°C. Arrows mark examples of large-budded cells used for Cdc42 quantification in B & C. Scale bar: 3 µm. **B.** Cdc42-mCherrySW levels in mother (m) and bud (b) compartments of large-budded ydj1Δ cells, grown at 27°C. (left plot) Mean fluorescence intensities from 19 representative mother-bud pairs are plotted. ns, p ≥ 0.05, paired *t* test. (right plot) The log2 mother-to-bud ratio of Cdc42-mCherrySW mean intensity in WT and ydj1 mutants (n = 52 per strain). The dotted line denotes a symmetric distribution of Cdc42 between mother and bud. **** p < 0.0001 by one-way ANOVA. See also S3D Fig. **C.** Cdc42 levels in WT and ydj1 mutants, grown at 27°C to mid-log phase. Mean fluorescence intensities of Cdc42-mCherrySW in whole cells (mother and bud combined) are plotted. n = 57 ~ 60 per strain; **** p < 0.0001, unpaired t-tests. Immunoblotting shows Cdc42-mCherrySW in each strain, detected using polyclonal anti-RFP antibodies, and α-tubulin, a loading control. See also S3C Fig. **D.** Association of Cdc42-mCherrySW with GFP-Ydj1 detected by a visible IP assay (top panel). A control reaction used extracts containing untagged Ydj1 (bottom panel). The data underlying the graphs can be found in S1 Data.
