# Supplementary figures and images for: Cdc42 interacts with chaperone Ydj1 to enhance its stability and partitioning during asymmetric cell division and aging in yeast
Source: PLoS Biol. 2026 Feb 12;24(2):e3003306. doi: 10.1371/journal.pbio.3003306 (PMC12900338; doi:10.1371/journal.pbio.3003306)

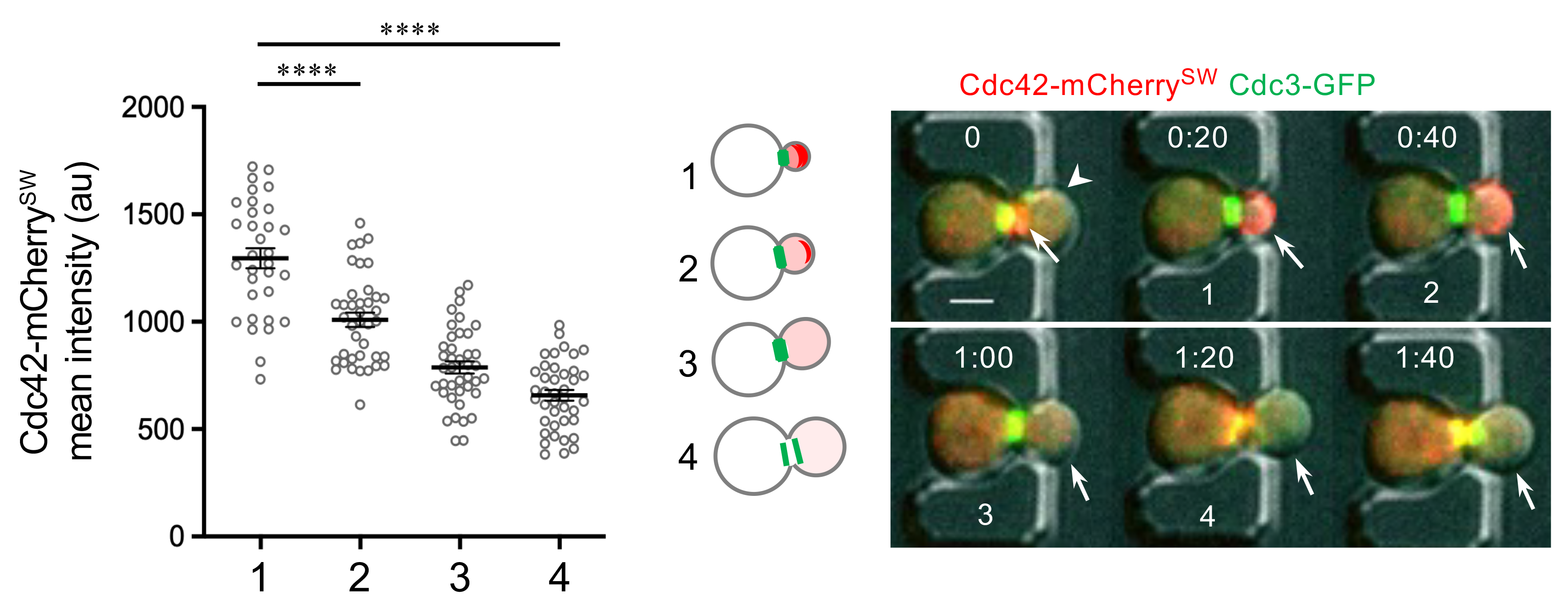

Supplement: S1 Fig — Mean fluorescence intensities of Cdc42-mCherrySW within growing buds are compared. A split septin ring (labeled with Cdc3-GFP) indicates the onset of cytokinesis. Arrows mark the same bud developing and producing a daughter; an arrowhead marks a daughter cell from the previous division. Scale bar: 3 µm. n = 32–40 per group; **** p < 0.0001, Welch’s t test. The data underlying the graphs can be found in S1 Data. (TIF) [file pbio.3003306.s001.tif]

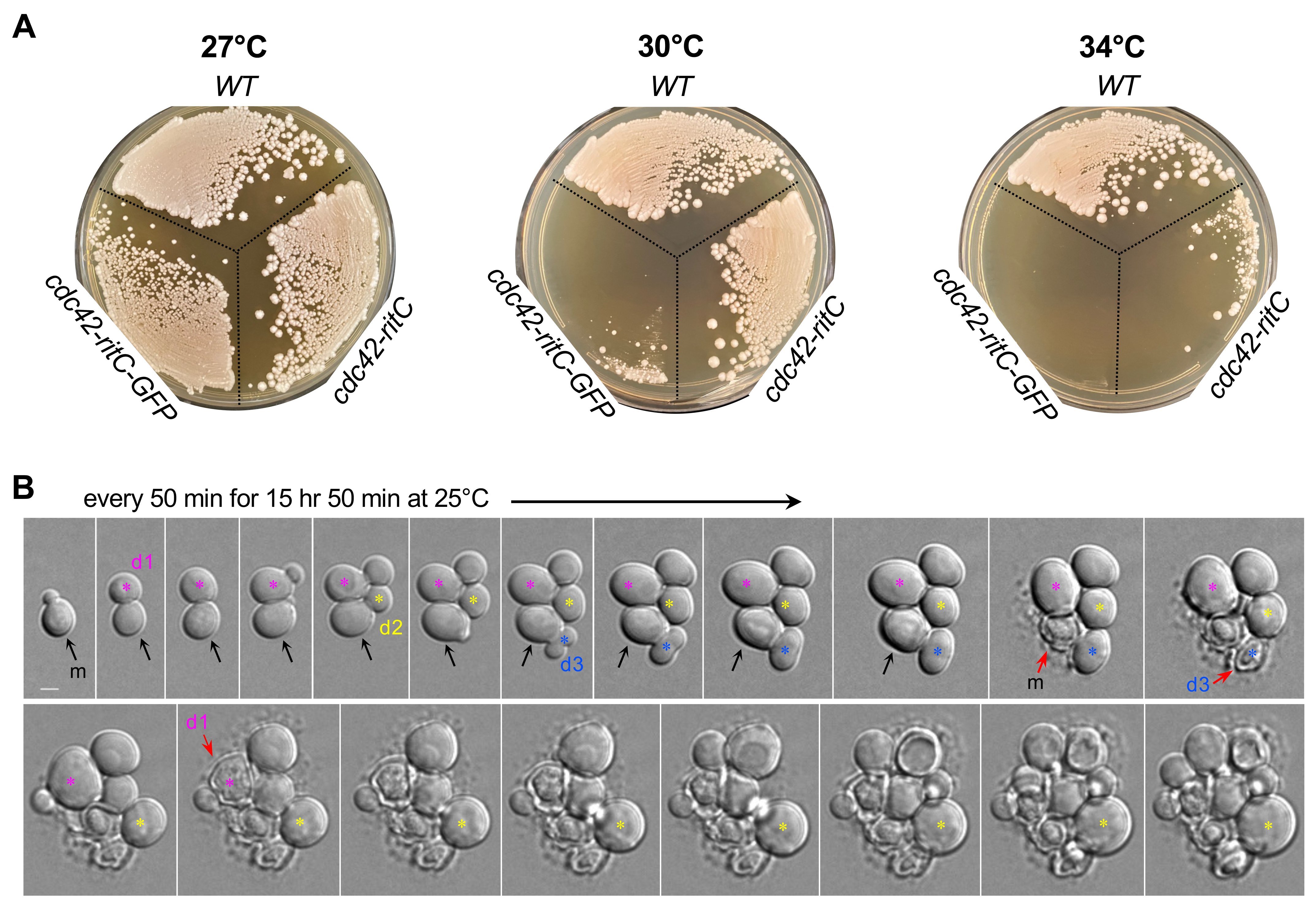

Supplement: S2 Fig — A. Growth phenotypes of cdc42-ritC, cdc42-ritC-GFP, and isogenic wild-type (WT) strains on YPD plates at 27, 30, and 34 °C. B. Time-lapse images of cdc42-ritC-GFP cells at 25 °C. Selected time points are shown every 50 min for about 16 hours. Colored asterisks mark daughter cells (d1–d3) originating from the same mother cell (black arrows). Red arrows denote cell death. Note: Daughter cell d2 continued to grow large without typical signs of cell death. See S2 Movie. (TIF) [file pbio.3003306.s002.tif]

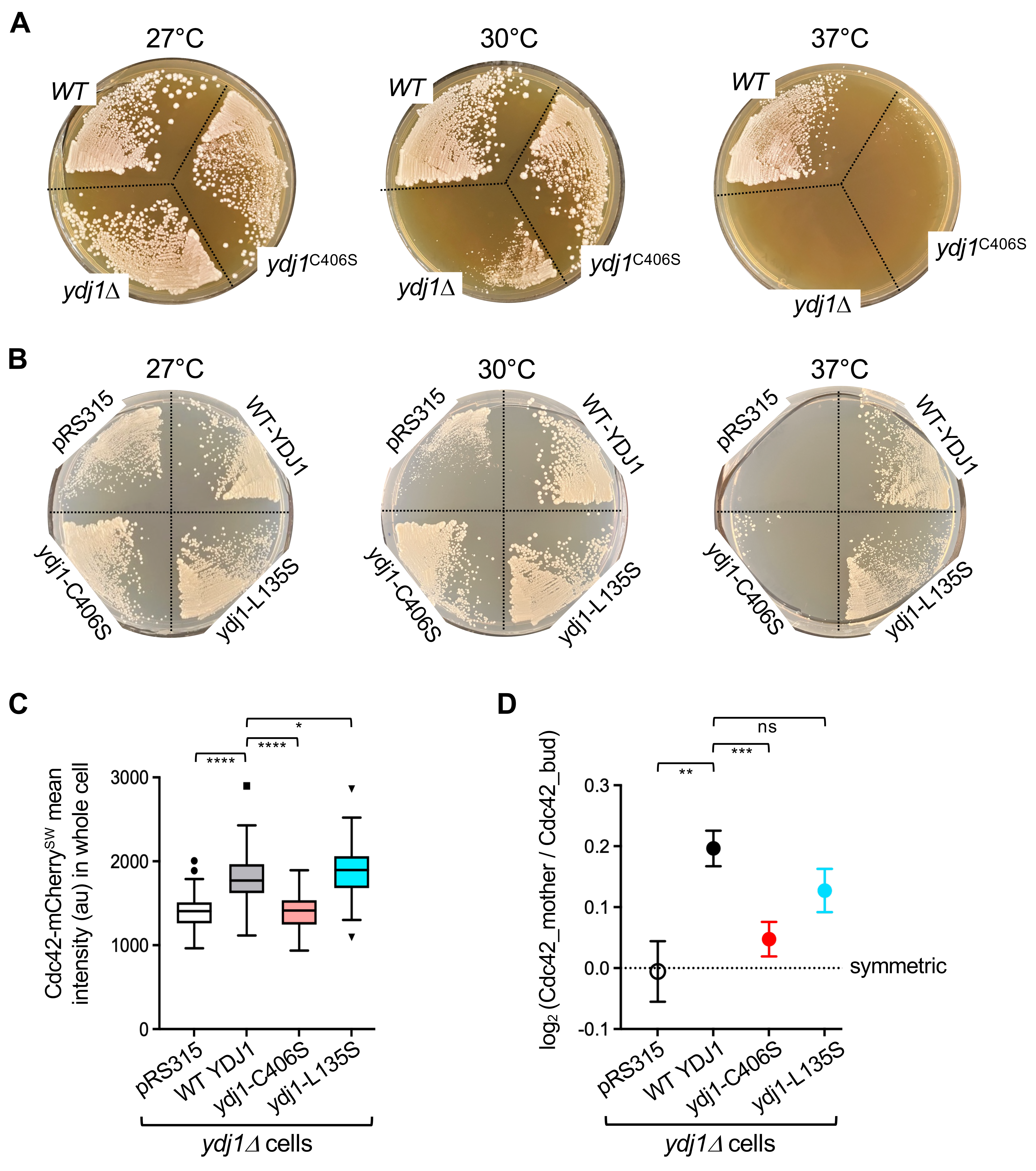

Supplement: S3 Fig — A. Growth phenotypes of ydj1Δ, ydj1(C406S), and isogenic WT strains on YPD plates at 27, 30, and 37 °C. B. Growth phenotypes of ydj1Δ cells carrying either WT YDJ1, ydj1(C406S), ydj1(L135S), or pRS315 plasmid on SC-Leu plates at 27, 30, and 37 °C. C. Mean fluorescence intensities of Cdc42-mCherrySW in whole cells (mother and bud combined) of the ydj1Δ CDC42-mCherrySW strain carrying each plasmid, grown at 27 °C (n = 78–86 per strain). **** p< 0.0001; *, p= 0.0333, by unpaired Welch’s t test. D. The log2 mother-to-bud ratio of Cdc42-mCherrySW mean intensity (mean ± SEM) in ydj1Δ cells carrying each plasmid, grown at 27 °C (n = 22–40 per group). p = 0.0005 by ordinary one-way ANOVA; ns, p ≥ 0.05; ** p< 0.01; and *** p< 0.001 by Welch’s t test. The data underlying the graphs can be found in S1 Data. (TIF) [file pbio.3003306.s003.tif]
