# Supplementary material for: Cdc42 interacts with chaperone Ydj1 to enhance its stability and partitioning during asymmetric cell division and aging in yeast
Source: PLoS Biol. 2026 Feb 12;24(2):e3003306. doi: 10.1371/journal.pbio.3003306 (PMC12900338; doi:10.1371/journal.pbio.3003306)
Supplement: S1 Table — (PDF) [file pbio.3003306.s006.pdf]

S1_Table. Yeast strains used in this study

Strain[a]	Relevant Genotype	Source/Comments
BY4741[@]	*MAT***a** *his3Δ1 leu2Δ0 met15Δ0 ura3Δ0*	Open Biosystems
YSC13[@]	*MAT*α *cdc42-ritC-kanMX*	[1]
YSC11[@]	*MAT*α *cdc42-ritC-GFP-kanMX*	[1]
DDY1300[@]	*MAT***a** *his3Δ200 leu2-3,112 lys2-801am ura3-52 CDC42:LEU2*	[2]
DDY1312[@]	*MAT***a** *cdc42-108*$^{R147A, E148A, K150A}$*:LEU2*	[2]
DDY4890[@]	*MAT***a** *CDC42-mCherry*^{SW}*:HIS3*	[3]
YWS2110[@]	*MAT***a** *his3Δ1 leu2Δ0 met15Δ0 ura3Δ0 ydj1*^{C406S}	[4]
HPY3740[@]	*MAT***a** *CDC42-mCherry*^{SW}*::HIS3 CDC3-GFP:LEU2*	This study
HPY3881[@]	*MAT*α *fob1Δ:: kanMX4*	Open Biosystems
HPY3963[@]	*MAT*α *cdc42-108:LEU2 fob1Δ:: kanMX4 met15 ura3 leu2*	Segregant of DDY1312 X HPY3881
HPY4079[@]	*MAT***a** *CDC42-mCherry*^{SW}*:HIS3 ydj1Δ:: kanMX4*	This study
HPY4092[@]	*MAT*α *CDC42-mCherry*^{SW}*:HIS3 ydj1*^{C406S}	Derived from YWS2110
HPY4148[@]	*MAT***a** *cdc42-108*$^{R147A, E148A, K150A}$*:LEU2 cdc42-108*$^{R147A, E148A, K150A}$*:URA3*	This study[b]
HPY210[#]	*MAT***a** *his3-Δ200 leu2-Δ1 lys2-801 trp1-Δ63 ura3-52*	[5]
HPY3721[#]	*MAT***a** *cdc42::TRP1 GFP-CDC42(8X)-URA3 CDC3-mCherry:LEU2*	[6]
HPY3215 [#]	*MAT***a** *cdc42::TRP1* P_{CDC42}*-GFP-CDC42:URA3*	Segregant of DLY13920 [7]

[a] Strains marked with [@] are derived from DDY1300 or BY4741 (both in S288C background); and strains marked with [#] are isogenic with HPY210, derived from YEF473 [8].

[b] A copy of *cdc42-108*$^{R147A, E148A, K150A}$ was obtained from DDY1312 by genomic PCR and cloned into pRS306. The resulting plasmid was integrated into the *URA3* locus of DDY1312 after digestion with *Eco*RV.

References for S1_Table

1. Bendezu FO, Vincenzetti V, Vavylonis D, Wyss R, Vogel H, Martin SG. Spontaneous Cdc42 polarization independent of GDI-mediated extraction and actin-based trafficking. PLoS biology. 2015;13(4):e1002097. Epub 2015/04/04. doi: 10.1371/journal.pbio.1002097. PubMed PMID: 25837586; PubMed Central PMCID: PMCPMC4383620.
2. Kozminski KG, Chen AJ, Rodal AA, Drubin DG. Functions and functional domains of the GTPase Cdc42p. Mol Biol Cell. 2000;11(1):339-54. doi: 10.1091/mbc.11.1.339. PubMed PMID: 10637312; PubMed Central PMCID: PMC14778.
3. Lu MS, Drubin DG. Cdc42 GTPase regulates ESCRTs in nuclear envelope sealing and ER remodeling. J Cell Biol. 2020;219(8):e201910119. Epub 2020/06/20. doi: 10.1083/jcb.201910119. PubMed PMID: 32556066; PubMed Central PMCID: PMC7401818.

4. Hildebrandt ER, Cheng M, Zhao P, Kim JH, Wells L, Schmidt WK. A shunt pathway limits the CaaX processing of Hsp40 Ydj1p and regulates Ydj1p-dependent phenotypes. eLife. 2016;5:e15899. doi: 10.7554/eLife.15899.
5. Singh K, Kang PJ, Park H-O. The Rho5 GTPase is necessary for oxidant-induced cell death in budding yeast. Proc Natl Acad Sci USA. 2008;105(5):1522-7. PubMed Central PMCID: PMCPMC2234177.
6. Kang PJ, Mullner R, Li H, Hansford D, Shen HW, Park H-O. Up-regulation of the Cdc42 GTPase limits the replicative life span of budding yeast. Mol Biol Cell. 2022;33(4):br5. Epub 2022/01/20. doi: 10.1091/mbc.E21-04-0208. PubMed PMID: 35044837; PubMed Central PMCID: PMC9250358.
7. Wu CF, Chiou JG, Minakova M, Woods B, Tsygankov D, Zyla TR, et al. Role of competition between polarity sites in establishing a unique front. eLife. 2015;4:11611. Epub 2015/11/03 06:00. PubMed PMID: 26523396; PubMed Central PMCID: PMCPMC4728132.
8. Bi E, Pringle JR. *ZDS1* and *ZDS2*, genes whose products may regulate Cdc42p in *Saccharomyces cerevisiae*. Mol Cell Biol. 1996;16:5264-75.
