# Supplementary material for: Cdc42 interacts with chaperone Ydj1 to enhance its stability and partitioning during asymmetric cell division and aging in yeast
Source: PLoS Biol. 2026 Feb 12;24(2):e3003306. doi: 10.1371/journal.pbio.3003306 (PMC12900338; doi:10.1371/journal.pbio.3003306)
Supplement: S1 Raw images — Full-size, uncropped, and labeled Western blots are shown for the indicated figure panels. The relevant strain genotypes, protein bands, and primary antibodies are labeled. Fig 1C. Cdc42 proteins in wild-type and cdc42–108 strains. Fig 3C. GFP-Cdc42 and Cdc42-ritC-GFP, expressed chromosomally in respective strains. Fig 4C. Cdc42-mCherrySW proteins in wild-type and ydj1Δ strains. (PDF) [file pbio.3003306.s007.pdf]

Fig. 1C

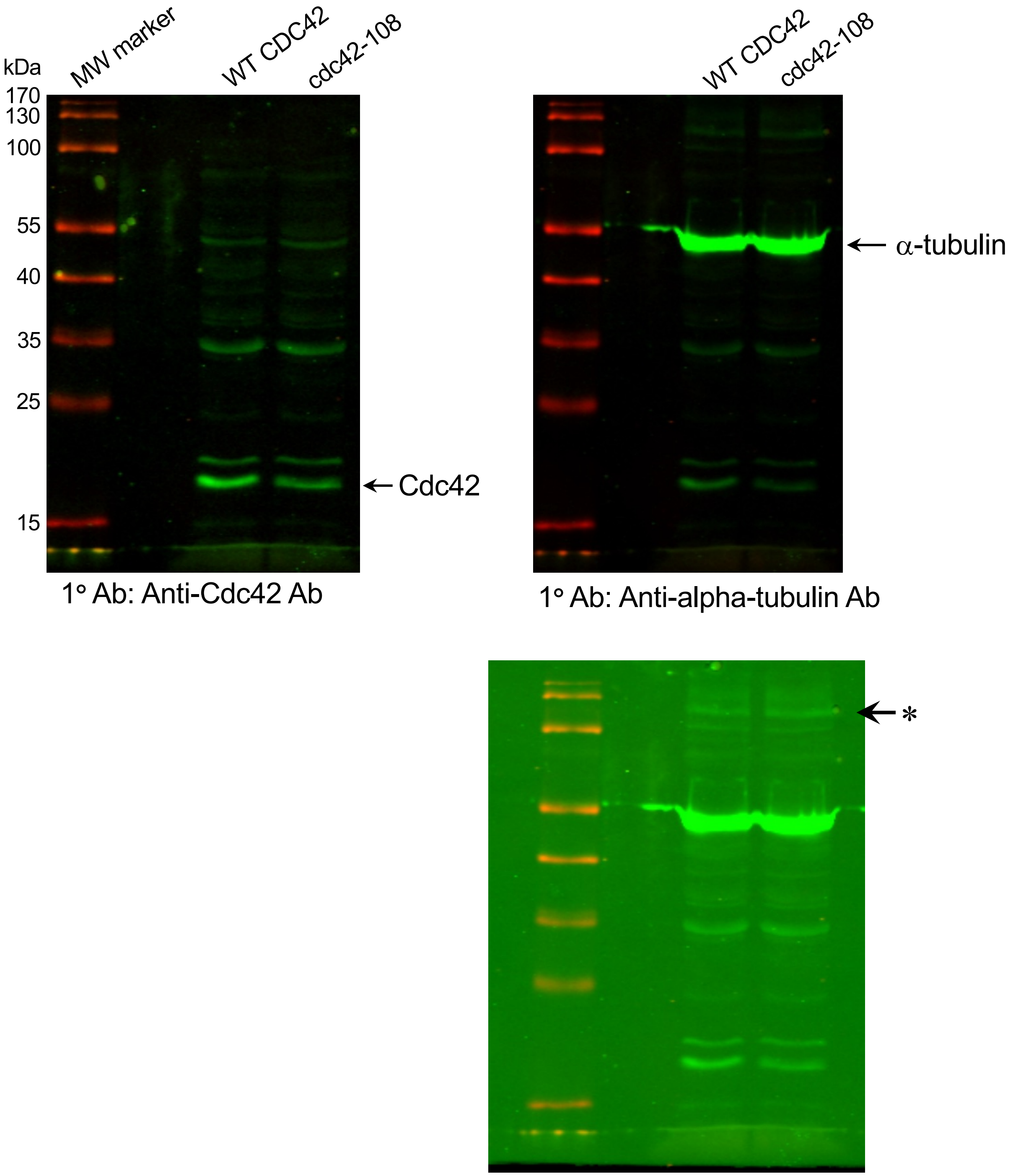

After PAGE using 12.5% gel, Cdc42 protein was detected with an anti-Cdc42 monoclonal antibody (left). As a loading control, α-tubulin was detected with an anti-α-tubulin monoclonal antibody (right). *Note*: A minor cross-reacting signal of Cdc42 appeared in the control blot because the same secondary antibody (IRDye800) was used to visualize a-tubulin after detecting Cdc42. The overexposed blot was used to quantify non-specific cross-reacting bands (e.g., marked with *) to confirm equal loading of the total cellular proteins in each lane and equal abundance of α-tubulin (a loading control) in each strain.

Fig. 3C

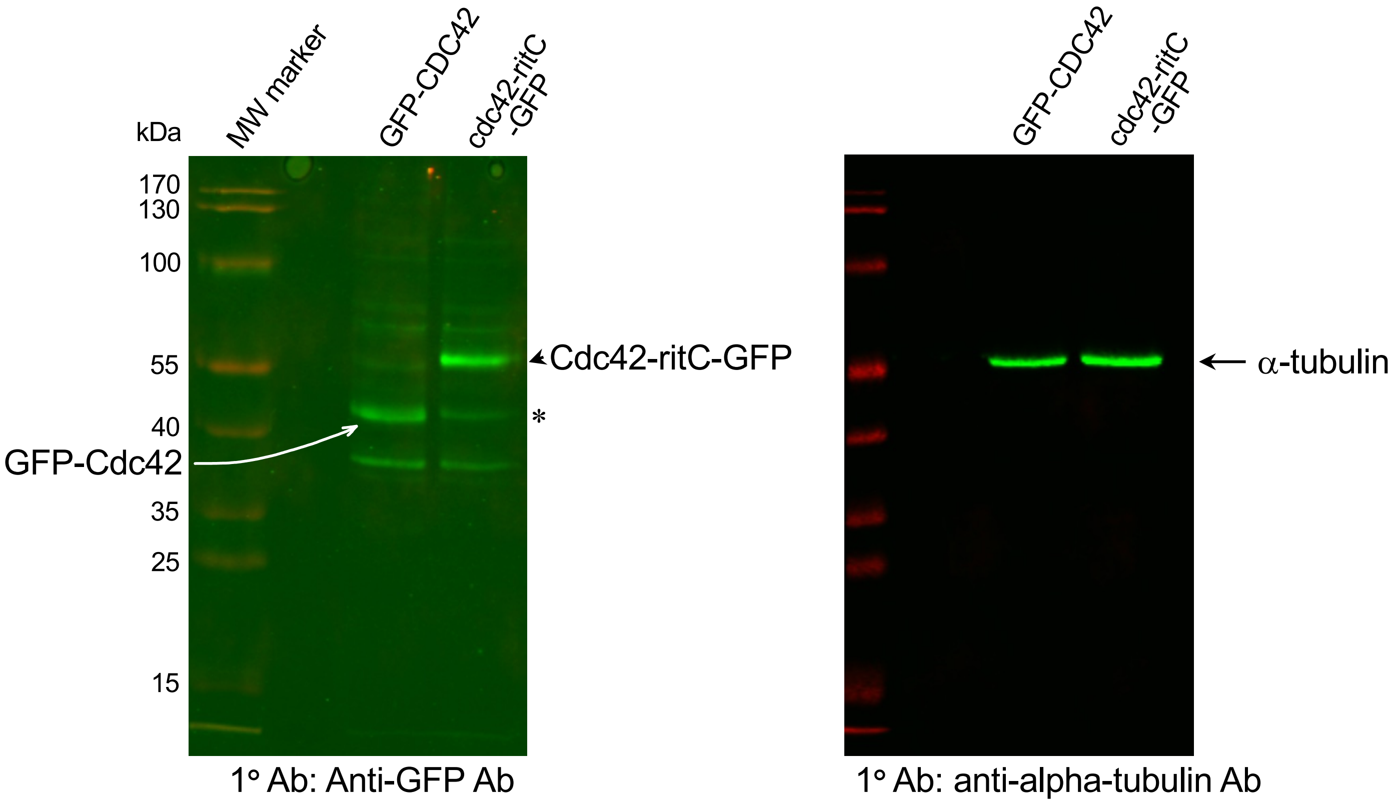

12.5% gel. GFP-Cdc42 and Cdc42-ritC-GFP, expressed chromosomally in respective strains, were detected using anti-GFP polyclonal antibodies (left), and α-tubulin was detected using anti-α-tubulin monoclonal antibody (right). *Note:* Because of some crossing-reacting proteins (*) with anti-GFP antibodies, these protein levels were also compared by quantifying GFP fluorescence in whole cells.

Fig. 4C

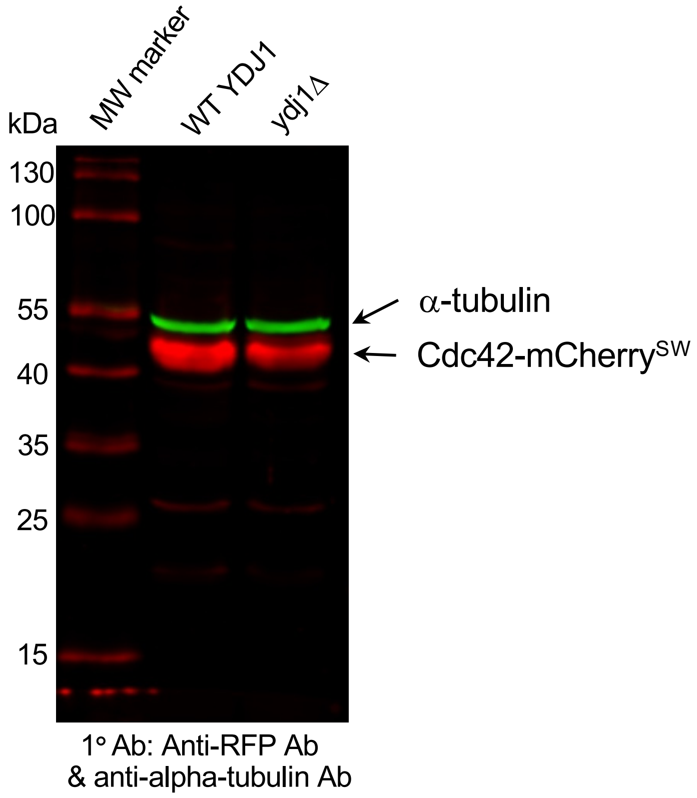

12.5% gel. Cdc42-mCherry^{SW} and α-tubulin were detected in wild-type and *ydj1*Δ strains using anti-RFP polyclonal antibodies and anti-α-tubulin monoclonal antibody, respectively.
