# Supplementary material for: Cdc42 interacts with chaperone Ydj1 to enhance its stability and partitioning during asymmetric cell division and aging in yeast
Source: PLoS Biol. 2026 Feb 12;24(2):e3003306. doi: 10.1371/journal.pbio.3003306 (PMC12900338; doi:10.1371/journal.pbio.3003306)
Supplement: Attachment — Submitted filename: Responses to Reviewers_121125.pdf [file pbio.3003306.s011.pdf]

PBIOLOGY-D-25-02023R1

Point-by-point responses to reviewers' comments
We sincerely appreciate the reviewers' thoughtful and valuable feedback on our manuscript. Below, we address each reviewer's comment *(shown in blue)*, followed by our detailed responses.

Reviewer 1:

The manuscript "Cdc42 Partitioning by Chaperone Ydj1 During Asymmetric Division and Aging in Yeast" by Kang et al. shows that Cdc42 partitions asymmetrically during growth and its association with the chaperone Ydj1 is important for this asymmetry that is has a role in cellular aging. The authors show that Cdc42 levels increase during aging, which is likely to be independent of rDNA silencing. Furthermore, they go on to show that this asymmetric partitioning of Cdc42 is not observed when this GTPase is constitutively targeted to the plasma membrane and that such asymmetric partitioning requires the farnesylated chaperone Ydj1. This brief report is clearly written, the data are convincing and the results are likely to be of interest to the broad audience of Plos Biology. There are some minor issues and details, which should be addressed to improve clarity and accessibility:

We thank the reviewer for these very positive comments and valuable feedback. We have addressed all minor issues, as described below.

Minor issues:
1) Some of the graph axes titles are unclear, including Fig. 1 'young and old ages' and Fig. 3A-C, in which it is not clear what the difference is between mother/daughter and young or old mother/daughter.

We apologize for the unclear labeling of the graph axes. Although the figure legends described 'young (first three divisions) and old ages (last three divisions) of mothers', we agree that clearly marking graph axes would improve clarity. The graph axes of all figures are revised accordingly, including '*young mother (first 3 divisions)*' and '*old mother (last 3 divisions)*' (**Fig. 1B)**; and '*young mother, daughter from young mother' and 'old mother, daughter from old mother*' (**Fig. 3A)**.

2) In the western blots, it is not clear that tubulin (which also may change with cell age) is the best control for normalizing protein levels. If other controls are not feasible this point should be mentioned.

Both immunoblots (Fig. 1C and Fig. 4C) compare Cdc42 levels in WT and mutant strains during exponential growth. Although we did not use an alternative antibody as a loading control, we normalized Cdc42 levels to total protein using Ponceau S-stained membranes. Additionally, we confirmed similar alpha-tubulin levels relative to total protein in both WT and mutant strains, using non-specific cross-reactive bands for reference (as shown in S1_raw images). This supports alpha-tubulin as a suitable loading control. We acknowledge the reviewer's concern about possible age-dependent changes in control protein abundance. However, this is unlikely to have affected our results, as all samples were collected at mid-log phase (OD_{600}

0.6–0.8) at permissive temperatures, when cultures primarily contain young, actively dividing cells. To address this, we have added further details on the growth conditions for protein preparation in the figure legends and Materials and Methods, as shown below:

- **Fig. 1C**. *Cdc42 proteins in WT and cdc42-108 cells, grown at 30°C to mid-log phase ...*
- **Fig. 3C**. *Levels of GFP-Cdc42 and Cdc42-ritC-GFP were compared by immunoblotting extracts from cells (expressing each allele as the sole genomic copy), grown at 25°C to early-log phase .....*
- **Fig. 4C**. *Cdc42 levels in WT and ydj1 mutants, grown at 27°C to mid-log phase.*
- *"WT and mutant strains were cultured in YPD medium at the temperatures specified in the figure legends and harvested at OD_{600} 0.6–0.8."*(**p. 11, lines 2–3**).

3) From Fig. 3 it is concluded that Cdc42 endomembrane association is critical, but another interpretation would be that association/dissociation from the membrane is what is critical and this should be mentioned.

We agree with the reviewer that this would be a more accurate interpretation. As suggested, we modified the text to: "*These results suggest that Cdc42's association with and release from internal membranes are crucial for its asymmetric distribution*." (**p. 5, lines 28–29)**

4) This is likely to be a semantic point; does lower levels of Cdc42 in daughters 'help them rejuvinate' (in abstract) or rather and more likely keep them young/slow down aging.

We thank the reviewer for this suggestion. It indeed seems inappropriate to use 'rejuvenate' in describing daughter cells, although such expression has been commonly used in previous publications. Accordingly, we modified the text where similar expressions were used, including: "*Daughter cells inherit lower levels of Cdc42, which likely helps them remain young*." (**Abstract, p. 2, lines 7–8**); "*These observations suggest that Cdc42 accumulates in mother cells during asymmetric division and that lower Cdc42 levels in daughters are likely to help maintain their youthfulness."* (**p. 5, lines 8–10**).

5) Is the effect of the ydj1 mutant (Fig. 4) due to a lack of an 'anchor' in the mother cell or an overall reduction of Cdc42 levels. The latter possibility seems to be a simpler explanation. This could be addressed by a repressible mutant or degron mutant, but I appreciate this is beyond the scope of this short report and hence should be mentioned.

While compartment-specific modulation of Cdc42 levels could be possible, as the reviewer noted, addressing this would require considerable additional work beyond the current revision. Although direct evidence is limited, our results suggest that the farnesylation-defective mutant *ydj1-C406S* is impaired in the asymmetric distribution of Cdc42 between mother and bud compartments during division (**Fig. 4B & S3_Fig, D**), as well as in maintaining Cdc42 protein levels (**Fig. 4C & S3_Fig, C**). Since Cdc42 asymmetry was evaluated by the mother-to-bud ratio of Cdc42 levels, the reduced asymmetric distribution in the *ydj1-C406S* mutant cannot be attributed solely to decreased overall Cdc42 levels (see below, #7). We also tested another *ydj1* mutant (*ydj1-L135S*), but it did not help further clarify the issues of this

asymmetry vs. stability. Because a single amino acid substitution impairing Ydj1's farnesylation affects both Cdc42's asymmetric distribution and its protein levels, these effects may not be easily separable.

Despite these limitations, we propose that Cdc42 is tethered to the endoplasmic reticulum of mother cells via Ydj1, resulting in its asymmetric distribution. This is supported by previous reports: farnesylated Ydj1 diffuses more slowly in mother cells than in their buds and is enriched in mother cells due to an ER diffusion barrier (Saarikangas et al., Curr Biol 2017, PMID 28262489), and the ydj1-C406S mutation affects the stability and maturation of Hsp90 clients, such as Ste11, whose interaction with Hsp90 depends on farnesylated Ydj1 (Flom et al., Mol Biol Cell 2008, PMID 18829866). We have included additional data, discussion, and limitations of this study (**p. 7, lines 3–14; p. 8, lines 1–7**).

6) It is unclear if cdc42-ritC is the sole copy of cdc42, if this is the case, does this strain have an aging defect, as would be expected?

Yes, *cdc42-ritC* is the only *CDC42* allele on the chromosome (see S1_Table). We have added additional characterization and description of this mutant, including the sentence "*Cdc42-ritC-GFP levels were comparable to GFP-Cdc42; both were expressed from the endogenous CDC42 promoter as the only genomic copy, indicating unaffected protein stability (Fig. 3C)*" (**p.5, lines 25–36**).

The *cdc42-ritC* mutant is likely short-lived, based on time-lapse imaging. Because of abnormal cell shapes or multi-budding phenotypes, it has been difficult to measure its lifespan by microfluidic imaging or micromanipulation. Nevertheless, time-lapse imaging revealed that newborn daughter cells frequently died only after a few cell divisions (**S2_Fig**; **Movie 2)**, suggesting a shortened lifespan of the *cdc42-ritC-GFP* strain. This finding is consistent with our suggestion that asymmetric distribution (and likely the lower levels) of Cdc42 during cell division is critical for establishing the full lifespan of newborn daughter cells. However, many sick strains also exhibit reduced lifespan even without directly impacting longevity. Both *cdc42-ritC* and *cdc42-ritC-GFP* strains exhibit poor growth compared to WT. These additional data and the caveats are discussed (**p. 5, line 23–p. 6, line 5)**. See also <u>Reviewer 2, #2</u> & <u>Reviewer 3, #1</u>.

7) In Fig. 4B, it seems that a ratio of mother/bud signals would be more appropriate than the bud value subtracted from the mother signal.

We are grateful to the reviewer for this suggestion. We agree with the reviewer that the ratio (Cdc42 fluorescence in mother/ Cdc42 fluorescence in bud) would be more reasonable in comparing Cdc42's asymmetric distribution among different strains, particularly because overall Cdc42 levels are different among these. We revised **Fig. 4B (right plot)** to represent the ratio after logarithmic transformation ($\log_2$), in addition to presenting the mean intensities in each compartment in representative mother-bud pairs of ydj1Δ cells (Fig. 4B, left). This ratio analysis confirms that the asymmetric distribution of Cdc42 is reduced in *ydj1Δ* and *ydj1-C406S* mutants ($p < 0.0001$, one-way ANOVA). Cdc42 asymmetry was also assessed similarly

in *ydj1Δ* cells transformed with each YDJ1 plasmid, including pRS315-ydj1-C406S and pRS315-ydj1-L135S, a mutation in the peptide-binding pocket (**S3_Fig, D**).

Reviewer 2:

This work reports that replicative lifespan in budding yeast depends on the asymmetric partitioning of Cdc42, a key regulator of cell polarity. Earlier findings suggested a connection between Cdc42 hyperactivation and premature aging in yeast. Here, the authors explore this connection using time-lapse fluorescence microscopy in microfluidic chambers, which allowed them to visualize the localization and accumultation of Cdc42 during numerous cell divisions of aging cells. They find that, although Cdc42 is enriched at the growing tips of daughter cells (bud) early in the division process, prior to cytokinesis Cdc42 becomes enriched in mother cells, and hence daughter cells inherit lower levels. Old mother cells accumulate increasing levels of Cdc42, suggesting that high levels may contribute to senescence and low levels in new daughter cells is rejuvenating. In support of this hypothesis, mutations in Cdc42 that reduce its levels confer increased lifespan. Further results show that the mother-bud asymmetry of Cdc42 depends on the ER-tethered chaperone protein, Ydj1, and that Cdc42 physically interacts with Ydj1 in cell extracts. Collectively, the findings reveal a surprising partitioning phenomenon for Cdc42 along with a potential underlying mechanism and physiological impact on yeast lifespan.

Overall, this manuscript provides thought-provoking findings and valuable new insights for the field. It fulfills the criteria for Short Reports format papers, in that it provides a concise presentation of novel and intriguing findings from a limited set of experiments. The experiments incisively discriminate among possible models, the data and analysis are high quality, and the interpretations are logical. I don't have any serious concerns or reservations about the work. As discussed in item #1 below, one minor weak point is the degree to which the level of Cdc42 (or the failure to partition it into the mother cell) can be regarded as a causal factor in aging. This is not a fatal flaw, but rather an area for potential improvement that might strengthen the overall conclusions.

We thank the reviewer for these very positive comments and thoughtful suggestions. We have addressed all specific points, as described below.

Specific points:

1. To address how increased Cdc42 levels might contribute to aging, the authors use a mutant allele, cdc42-108, to reduce Cdc42 levels by roughly a factor of 2. The results clearly show that this allele increases lifespan (Figs 1D, 2B), which is consistent with the hypothesis that elevated Cdc42 levels play a causal role in aging. This is a reasonable interpretation. However, an alternative hypothesis is that the triple mutations in this variant protein (R147A E148A K150A) disrupt some unknown function that promotes senescence. A possible way to distinguish between these alternatives would be to introduce a second genomic copy of the cdc42-108 allele. If the 1x mutant allele conferred a longer lifespan than the 2x mutant allele (which might show a similar lifespan as the WT allele), it would more convincingly argue that the lifespan difference is

dictated by different levels rather than by different functionality. Such an approach could strengthen the causality argument, but it is not essential.

We are grateful to the reviewer for suggesting this critical test. As suggested, we introduced an extra copy of *cdc42-108(R147A E148A K150A)* into the *cdc42-108* mutant, generating a strain with two copies of this allele on the genome ('2 x cdc42-108' = HPY4148). We compared its lifespan to the original *cdc42-108* mutant and the isogenic wild-type strain by pairwise microfluidic imaging (WT vs. HPY4148; and *cdc42-108* vs. HPY4148). These assays confirmed that HPY4148 has a shorter lifespan than *cdc42-108*. A few parallel microfluidic imaging sets of WT and HPY4148 confirmed no significant difference between WT and the *2x cdc42-108* strain (**Fig. 1E**). This shows that lower Cdc42 protein levels, rather than loss of function, account for the extended lifespan of *cdc42-108*, further supporting our conclusion that Cdc42 elevation during aging limits lifespan (**page 4, lines 7–11**).

2. The authors did not report whether the Cdc42-ritC derivative, which disrupts the asymmetry in mother-bud distribution, had an impact on lifespan. This could potentially address whether the normal asymmetric partitioning has a causal effect on aging. Conceivably any interpretaion could be confounded if the ritC fusion substantially alters total Cdc42 protein levels. It was not clear if the results address this, as the fluorescent tag is different for Cdc42-ritC (GFP) than for other experiments (mCherry). Some direct commentary on this issue seems worthwhile.

We agree with the reviewer that directly testing the causal effects of Cdc42's asymmetric partitioning versus its levels on aging would be invaluable. As discussed above (<u>Reviewer 1, #6</u>), we performed additional characterization of the *cdc42-ritC* mutant. The total cellular levels of Cdc42-ritC-GFP and GFP-Cdc42 (both expressed from the endogenous *CDC42* promoter as the sole genomic copy) were not significantly different (**Fig. 3C**), suggesting that replacing the CaaX motif with the hydrophobic tail of Rit does not affect overall protein stability. We also found that the *cdc42-ritC* mutant has a notably short lifespan (**S2_Fig**; **S2_Movie)**, which supports the idea that reduced Cdc42 levels in daughter cells are important for retaining full lifespan. However, the complex phenotypes of the *cdc42-ritC-GFP* mutant make it difficult to directly link its reduced lifespan to the protein's symmetric distribution. This is also because many mutants with growth defects exhibit shortened lifespans, even without directly affecting longevity. Nevertheless, we have included relevant data and discussed these caveats in response to all reviewers' comments (**p. 5, line 23–p. 6, line 5**). See also <u>Reviewer 1, #6</u> & <u>Reviewer 3, #1.</u>

3. Page 5: Regarding Fig 4C, the authors state that "Cdc42-mCherrySW fluorescence was diminished in both mother and bud compartments of ydj1 mutant cells". The data don't seem to support this statement. Namely, the statement seems to imply that the fluorescence was measured separately in both mother and bud, and that the levels were reduced in each compartment. But Fig 4C does not analyze mother and bud separately, and instead measures them combined together (from the legend: "...intensities in the whole cells [including both mother and bud compartments]..."). I suspect the confusion is unintentional, and perhaps it could be fixed simply by rewording the main text to say "...the total cellular Cdc42-mCherrySW fluorescence (mother and bud combined) was diminished in ydj1 mutant cells".

We thank the reviewer for noticing this confusing sentence. Indeed, in **Fig. 4C**, Cdc42 levels between WT and *ydj1* mutants were compared by quantifying the mean fluorescence intensity of Cdc42-mCherry^{SW} in entire cells (mother and bud combined) and by immunoblotting. The text has been modified, as suggested, but avoided using the term 'total fluorescence' (as it can be confused with total pixel intensity, i.e., IntDen). We analyzed mean pixel intensity in this analysis to exclude any potential variability (such as cell size) due to the mutation.

The revised text now states: " *Additionally, Cdc42 protein levels were reduced in ydj1Δ and ydj1(C406S) mutants compared to WT, as shown by Cdc42-mCherry^{SW} fluorescence analysis in whole cells and by immunoblotting ......*" (**p. 6, lines 23–25**).

Reviewer 3:

In this report the authors show that Cdc42 asymmetrically distributed in aging cells and enhanced Cdc42 levels is associated with replicative aging. This is an important observation since enhanced Cdc42 levels has been observed in different aged cells types. Increased Cdc42 levels have been shown to shorten life span right from yeast to animals. However, it is not clear how this is mediated at the molecular level. The authors show that the asymmetric distribution is dependent on farnesylated Ydj1, an Hsp40/DnaJ chaperone tethered to the endoplasmic reticulum. While this is an interesting observation, it is too preliminary in its current form. Additional characterization of this observation will make these findings more convincing. The major issues are mentioned below.

We appreciate the reviewer's recognition of the importance of our findings and valuable feedback. To address these comments, we performed additional experiments for this revision. While more detailed mechanistic studies will be pursued in the future, we have responded to each point below.

Major points
1. The authors show that Cdc42-RitC fails to asymmetrically distribute between the two daughter cells. If asymmetric Cdc42 distribution continues to aging, these cells should not age as rapidly. How do the Cdc42-RitC cells age? Given that Cdc42-RitC is remains in the plasma membrane, if the cells age slowly then it will indicate that the enhanced endomembrane Cdc42 is needed for aging. If they age the same as control cells then only the overall Cdc42 levels matter and not its location.

As discussed above (Reviewer 1, #6; Reviewer 2, #2), the *cdc42-ritC-GFP* strain likely has a short lifespan. Although directly assessing its lifespan is challenging, we found that most daughter cells are short-lived (**S2_Fig & Movie 2**). Overall cellular levels of Cdc42-ritC-GFP and GFP-Cdc42 are similar, as both are expressed from a single genomic copy of their respective alleles (**Fig. 3C**). However, symmetric distribution leads to daughter cells being born with high Cdc42-ritC-GFP levels at division (**Fig. 3D**)—much like the daughters of old wild-type mothers undergoing symmetric division (see Fig. 3B)—and this likely contributes to a shortened lifespan. Nevertheless, due to the complexity of the *cdc42-ritC* mutant phenotype and the potential involvement of multiple factors, it is difficult to determine precisely how

much of the lifespan reduction is due to loss of asymmetric distribution or loss of Cdc42's endomembrane association. We have discussed these limitations and caveats (**page 5, line 30– page 6, line 5**).

2. How do ydj1 cells age? Do they show a phenotype similar to increased life span in fob1 mutants. Would a fob1ydj1 show an enhanced life span while the ydj1cdc42-108 double mutant would be epistatic? If ydj1 cells age slowly, can this be rescued with Cdc42 overexpression?

If Ydj1's only function was to maintain Cdc42 protein levels, we would expect *ydj1*Δ mutants to live longer than wild type due to reduced Cdc42 levels. However, Ydj1 is critical for protein quality control, and so Ydj1-deficient cells have a reduced replicative lifespan compared to WT (Hill et al, Science 2014, PMID 24855027). Ydj1/Hsp40 works with other Hsps to maintain protein homeostasis and manage misfolded or aggregated proteins as cells age (Moreno et al., eLife 2019, PMID 31518229). Ydj1 is also involved in the asymmetric segregation of protein aggregates during division (Saarikangas et al., Curr Biol 2017, PMID 28262489). Given Ydj1's various roles in proteostasis, we think lifespan analyses of *ydj1*Δ combined with *cdc42* variants or *fob1*Δ are unlikely to provide clear insights into its relationship with Cdc42.

3. The enhanced Cdc42 in aged cells could be due to a stress response similar to that observed in cells at high temperature. While the authors present data that ydj1 mutants show decreased levels of Cdc42 at low temperatures, that fact that these mutants show enhanced CDc42 levels at high temperature is confusing and needs further investigations. Is it possible to make a ydj1hsp90 double mutant? Would this cell show diminished level of Cdc42 even at high temperatures.

As the reviewer noted, elevated Cdc42 levels in aged cells may partially result from stress responses. Indeed, many studies suggest a complex interplay between cellular stress response and aging. However, currently available data do not clearly connect elevated Cdc42 to the stress response in yeast. While increased and hyperactive Cdc42 has been observed in aging yeast and metazoans by our group and others (Florian et al., Cell Stem Cell 2012, PMID 22560076; Florian et al., Aging Cell 2020; Nalapareddy et al., iScience 2021, PMID 33870147; Kang et al., Mol Biol Cell 2022, PMID 35044837; and Kang et al., this study), a previous study reported that Cdc42 is degraded by Hsp40-Hsp70 at 37°C in WT cells (González et al., Mol Cell Biol 2023, PMID 37114947).

As the reviewer mentioned, the contradictory effects of *ydj1*Δ on Cdc42 levels observed by our study and by González et al. at different temperatures are puzzling and need further examination. While we are confident in our data, we have some concerns regarding the other group's results at 30°C or at 37°C (González et al., J Cell Biol 2022, PMID 36350310; MCB 2023, PMID 37114947) for two main reasons:

1) They cultured their *ydj1*Δ mutant at 30°C and then shifted to 37°C for a few hours. However, *ydj1*Δ mutants grow poorly at 30°C and cannot grow at 37°C (see **S3_Fig**), as also reported by others (Caplan et al., J Biol Chem 1992, PMID 1527016; Atencio & Yaffe, Mol Cell Biol 1992,

PMID 1729605; Flom et al., Mol Biol Cell 2008, PMID 18829866). Oddly, they stated that "cells lacking Ydj1p or Ssa1p did not show a growth defect," and their serial dilutions showed no growth differences between WT and *ydj1*Δ (González et al., PMID 36350310). These raise concerns about their strain authenticity.
2) They used GFP-Cdc42 to analyze protein levels and localization at 30°C or 37°C. However, GFP-Cdc42 is only partially functional: strains with *GFP-CDC42* (replacing endogenous *CDC42)* grow poorly at 30°C and are inviable at 37°C (Wu et al. eLife 2015, PMID 26523396), raising some concerns about using non-functional GFP-Cdc42 at high temperatures.

Nevertheless, we provided a possible explanation for these different observations on **page 7 (lines 16–23).** It is known that at 30°C or above *, ydj1*Δ mutants have impaired growth and trigger a stress response that increases Hsp70 and Hsp90 expression (Caplan et al., PMID 1527016; Atencio & Yaffe, PMID 1729605; Mandal et al., MCB 2008, PMID: 18443039). Thus, the increased Cdc42 observed in *ydj1Δ* mutants under these conditions might reflect indirect compensatory mechanisms rather than direct effects of losing Ydj1. Indeed, our data suggest chaperons are likely involved in Cdc42 folding or stability, and this deserves further study, especially of Hsp90, as the reviewer suggested. However, we believe further clarifying *ydj1*Δ's effects on Cdc42 at elevated temperatures or examining genetic interactions with hsp90 is beyond the scope of this brief report.

Minor points

1. The authors state that the fob1⍰ mutant is long lived. However, the survival curve of fob1⍰ cells in fig2B show shorter live span that the WT cells in fig 1D.

The *fob1*Δ mutant is a well-established, long-lived yeast strain, with its extended lifespan associated with decreased ERC formation (Defossez et al., Mol Cell. 1999, PMID 10230397). While the extent of lifespan extension can vary depending on genetic background, our microfluidic imaging-based assay showed that the *fob1*Δ mutant had a median lifespan of 19 generations (**Fig. 2B**), compared to 17 or 18 generations for the WT strain (**Fig. 1D**). This represents an approximately 11% increase in median lifespan for *fob1*Δ in this genetic background. Our lifespan estimation is based on tracking cell divisions via microfluidic imaging, which can occasionally miss extremely long-lived cells due to rare events like cell loss or crowding at later stages. In addition, not all cells loaded in microfluidic chambers are newborn daughter cells, unlike micromanipulator-based assays (which start with newborn daughter cells or spores). For these reasons, we emphasize relative median lifespan between strains, which is highly reproducible, particularly when multiple strains are imaged in parallel using chambers containing several flow passages.
