# Supplementary material for: Cdc42 interacts with chaperone Ydj1 to enhance its stability and partitioning during asymmetric cell division and aging in yeast
Source: PLoS Biol. 2026 Feb 12;24(2):e3003306. doi: 10.1371/journal.pbio.3003306 (PMC12900338; doi:10.1371/journal.pbio.3003306)
Supplement: Attachment — Submitted filename: Responses to Reviewer 3.docx [file pbio.3003306.s012.docx]

PBIOLOGY-D-25-02023R2

Below, we respond to *Reviewer 3’s comments* (*in italics*) and outline our manuscript updates.

*The authors claim in a subtitle that "Cdc42 is asymmetrically distributed during cell division through its association with endomembranes". However, this statement has not been tested. What the authors do show is that stably restricting Cdc42 to the plasma membrane (Cdc42-RITC) leads to symmetric Cdc42 distribution between mother and daughter buds. It is not shown whether this symmetry is due to the lack of Cdc42 dynamics at the plasma membrane, the lack of exchange with the GDIs or the lack of localization to the endomembrane.*

**–** The subtitle makes two points: (1) Cdc42 is asymmetrically distributed during cell division, and (2) this asymmetry arises through its association with endomembranes. Our study directly demonstrates the first point, while the second is indirectly supported. Although we have not explicitly tested this, specific properties of Cdc42-ritC, noted by the reviewer, are unlikely to account for its symmetric distribution, as discussed below.

**1. Diffusion:** Cdc42-ritC shows slower lateral diffusion at the plasma membrane than wild-type Cdc42, as shown in our previous work (Kang et al. 2018, Mol Biol Cell, PMID: 30091649). However, we believe that reduced mobility cannot explain symmetry; if diffusion were the limiting factor, the newly formed buds (and daughter cells) would likely have **lower Cdc42 levels**. Instead, we observe symmetric distribution of Cdc42-ritC, indicating that the mutant protein efficiently reaches the daughter cell.

**2. GDI:** Cdc42-ritC is thought not to interact with GDI, because it lacks the prenyl group. GDIs extract Rho GTPases from membranes (including the plasma membrane), keeping them inactive and soluble in the cytosol. Proteins freely floating in the cytosol tend to equilibrate quickly between mother and bud. Similarly, wild-type Cdc42 in soluble GDI-bound pools is unlikely to drive asymmetric partitioning. Known mechanisms of asymmetric partitioning (such as protein aggregates) typically involve membrane association, membrane-associated septin-dependent diffusion barriers, or organelles (Yang et al. 2015, PNAS, PMID: 26351681; Zhou et al. 2011, Cell, PMID: PMID22118470). Therefore, the impaired GDI interaction of Cdc42-ritC is unlikely to influence its distribution between mother and daughter cells.

*Moreover, that fact that Cdc42 levels drop in ydj1 mutants indicate that asymmetry could be either due to Ydj1 mediated Cdc42 stability or due to Ydj1 anchoring Cdc42 to the ER. Thus the claim that endomembrane associated Cdc42 leads to asymmetry is not substantiated.*

**–** This issue (stability vs. distribution) was addressed in detail in our previous response (see points #5 and #7 to Reviewer 1) and in the revised manuscript. We showed that the farnesylation-defective *ydj1-C406S* mutant, which is impaired in ER tethering, specifically fails to establish asymmetry. This defect cannot be explained by an overall reduction in protein levels, because asymmetry is quantified as **the mother-to-bud ratio**. These findings are consistent with prior work showing that farnesylated Ydj1 diffuses more slowly in mother cells than in buds (Saarikangas et al., 2017, Curr Biol, PMID: 28262489). Based on these, we propose that farnesylated Ydj1 promotes the asymmetric distribution of Cdc42.

**Manuscript Updates**

To address the reviewer’s concerns about the limited direct evidence, we have made the following revisions (see pages 5 and 6):

- We changed the subheading to: “Cdc42 is asymmetrically distributed during cell division, **likely** through its association with, and release from, cellular membranes”.
- We clarified in the text that we currently cannot determine whether Cdc42’s association with internal membranes or its release from membranes is critical for its asymmetric distribution.
